# Exploring the promising application of Be_12_O_12_ nanocage for the abatement of paracetamol using DFT simulations

**DOI:** 10.1038/s41598-023-45674-3

**Published:** 2023-10-28

**Authors:** Sana Gul, Kainat, Qaisar Ali, Momin Khan, Munir Ur Rehman, Abdullah F. AlAsmari, Fawaz Alasmari, Metab Alharbi

**Affiliations:** 1 Department of Chemistry, Abdul Wali Khan University Mardan, Mardan, 23200 Pakistan; 2 Heilongjiang Provincial Key Laboratory of CO2 Resource Utilization and Energy Catalytic Materials, School of Material Science and Chemical Engineering, Harbin University of Science and Technology, No. 4, Linyuan Road, Harbin, 150040 People’s Republic of China; 3 Department of Pharmacology and Toxicology, College of Pharmacy, King Saud University Riyadh, 11451 Riyadh, Saudi Arabia

**Keywords:** Computational biology and bioinformatics, Energy science and technology, Materials science

## Abstract

The removal of paracetamol from water is of prime concern because of its toxic nature in aquatic environment. In the present research, a detailed DFT study is carried out to remove paracetamol drug from water with the help of Be_12_O_12_ to eliminate the related issues. Three different geometries (CMP-1, CMP-2, CMP-3,) are obtained with the highest adsorption energies value (*E*_ads_) of − 31.2316 kcal/mol for CMP-3 without any prominent structural change. It is observed from the study that O atom from the carbonyl group (C=O) and H atom from O–H group successfully interact with O and Be atoms of the nanocage respectively. Natural bonding orbitals analysis reveals charge transfer to paracetamol drug from Be_12_O_12_ nanocage with maximum charge transfer of − 0.159 e for CMP-3 with bond angle of 1.65 Å confirming the stability of the CMP-3 among the optimized complexes. The quantum theory of atoms in molecule concludes that the interaction between paracetamol drug molecule and Be_12_O_12_ is purely closed-shell weak electrostatic in nature in CMP-1 and CMP-3 and shared interaction in CMP-2. The thermodynamics analysis witnesses that the process is exothermic and spontaneous. The regeneration study reveals the reversible nature of the adsorbent. The overall study presents Be_12_O_12_ nanocage as a potential adsorbent and may be used in future for the purification of water from a number of emerging pollutants.

## Introduction

The advancement and increase in sensitivity of modern analytical instruments has made possible the recognition of a growing number of novel organic substances in groundwater, natural water and wastewater. Particularly, the modern research has focused special attention on emerging pollutants such as personal care products and pharmaceuticals that are unable to be treated by commonly used wastewater treatment plants (WWTPs) and demand alternative and advance treatment processes. Numerous studies have shown the presence of pharmaceuticals in effluents and surface water affected by effluents from WWTPs^1–5^. Several other studies reported the existence of pharmaceuticals in drinking and groundwater^6^. Similarly, sea water is also polluted by the discharge of chemical products, cosmetic material usage, extensive consumption of medicines and discharge from industries which include pesticides, phenols, pharmaceuticals and organic dyes. These pollutants are toxic towards the ecosystem even in small concentration and adversely affect human’s health^7^.

Pharmaceuticals are used to cure diseases. The extensively used pharmaceuticals for the treatment of animal and human diseases are nonsteroidal anti-inflammatory drugs (NSAIDs) due to their antipyretic, anti-inflammatory and analgesic actions^8^. The excessive usage of NSAIDs for human and animal diseases contaminates water which adversely affects human and animal health^9,10^. Currently, different techniques are applied to eliminate NSAIDs from water which include photodegradation^11^, flocculation-coagulation^8^, biodegradation^9^, chlorination^10^, advance oxidation and ozonation^12,13^. However, the aforementioned methods are limited due to low efficiency, high cost and adverse environmental effects. Adsorption is the most effective method for the removal of these pollutants^14,15^.

Paracetamol (*N*-(4-hydroxyphenyl)acetamide), commonly recognized as acetaminophen, is the most extensively used antipyretic and analgesic throughout the globe. It has been detected in drinking water, wastewater and surface water because of its high solubility, stability and hydrophilicity^16,17^. The existence of paracetamol in wastewater has been reported previously up to 200 μg/L and 28.70 μg/L in surface water respectively^18^, despite of its high removal capacity about 90% in WWTPs^5,19,20^.These processes are however not enough for the complete removal of NSAIDs and a number of these contaminants persist without any change after treatment. Adsorption is the mostly employed method with high efficiency and promising results for the abatement of emerging pollutants due to cost effectiveness, possibility of reuse and regeneration, application at very low concentration and simple operation. Different 2D and 3D adsorbents have been used for elimination of pharmaceuticals from water^21^. Nanostructure materials possess several desirable properties and have been applied for a number of applications^22–24^. Carbon based nanotubes, single and multiwall carbon nanotubes, chitosan grafted graphene oxide composites, graphene, biochar, TiO_2_/GO composites, graphene oxide and magnetic mesoporous carbon have been applied for the adsorption of pharmaceuticals with good results^25–28^. However they are limited by long term sustainability and high toxicity^29–33^. Recently, boron nitride and aluminium nitride and other nanomaterials have been present as the most suitable adsorbents for the abatement of large number of pollutants from wastewater due to their high hydrophobicity, high thermal and structural stability, resistance to oxidation and wide bandgap^34–41^. Similarly, X_12_Y_12_ type inorganic nanocages such as B_12_N_12_ and Al_12_N_12_ have been explored for the removal of pollutants due to possessing distinctive physical, chemical and surface properties^42–51^. X_12_Y_12_ type inorganic nanocages have been used theoretically for the removal of paracetamol^52^ and experimentally for the adsorption of aspirin^53^ and for the sensing of toxic gases like ClCN, NH_3,_ and HCN^54,55^. Moreover, Al_12_P_12_ nanocages were examined for sensing of CH_3_F and CH_3_Cl efficiently^56^.

Considering the distinctive properties and promising results of X_12_Y_12_ type inorganic nanocages, we examined Be_12_O_12_ nanocage for the adsorption of paracetamol from wastewater computationally. The interaction between drug and nanocage was studied using bond length and adsorption energies analyses. The results were further confirmed by using DFT tools such as natural bonding orbital (NBO), reduced density gradient (RDG), non-covalent interactions (NCI), electron density difference (EDD), dipole moment (DM), frontier molecular orbital (FMO), and quantum theory of atoms in molecule (QTAIM) analyses. The results presented Be_12_O_12_ nanocage a promising adsorbent for the removal of paracetamol from wastewater.

## Results and discussion

### Optimization of monomers, complexes, reduced density gradient (RDG) investigation and non-covalent interaction (NCI)

**Figure 1:**
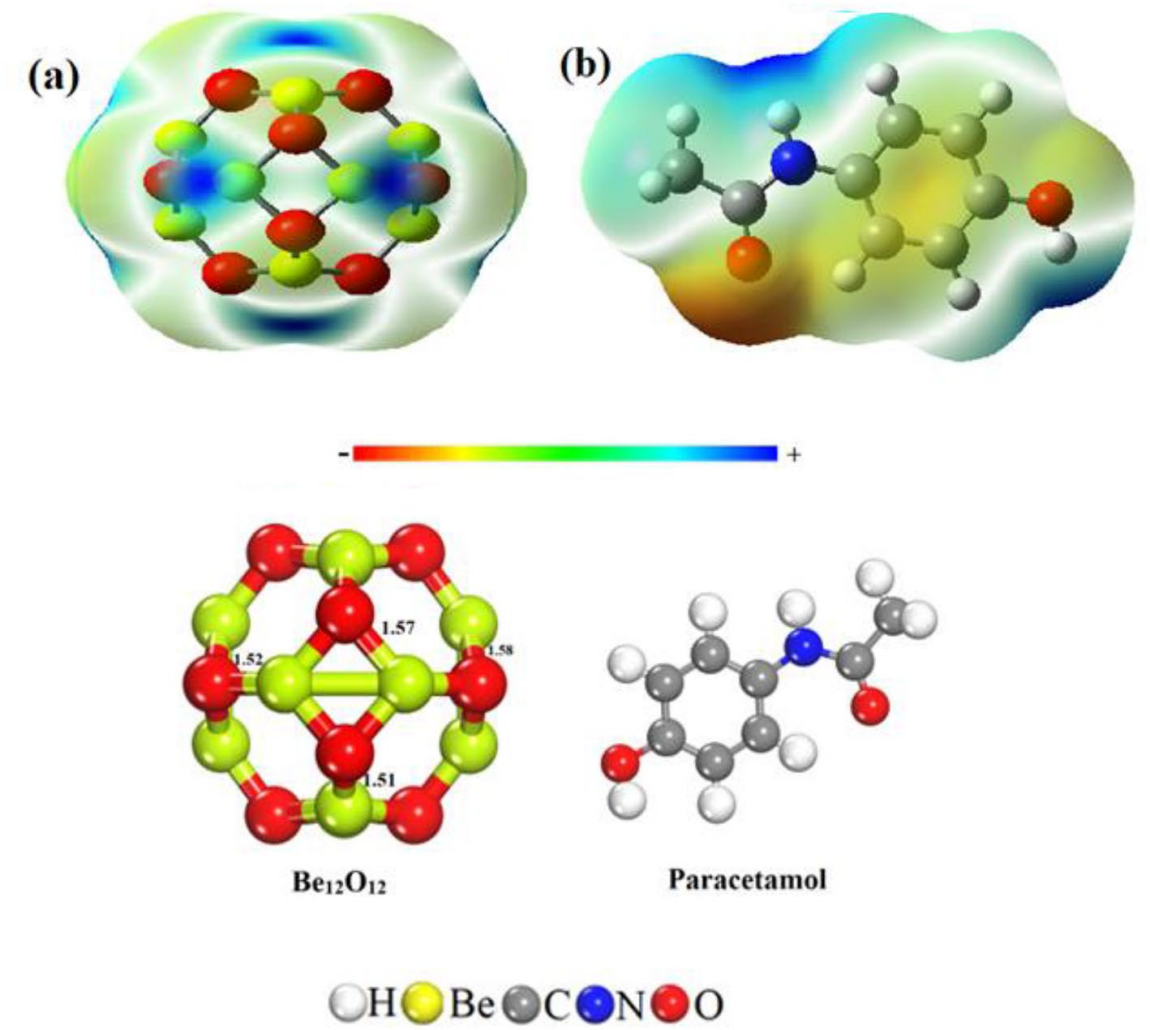
MEP map and optimized geometries of Be_12_O_12_ nanocage and paracetamol.

Density functional theory (DFT) simulations utilizing the B3LYP/6-31G(d,p) basis set were employed to optimize the Be_12_O_12_ nanocage. DFT is a successful theory to simulate the electronic structure and properties of molecules. The structure of the nanocage includes 6 four-membered rings (4MRs) and 8 six-membered rings (6MRs), as depicted in Fig. 1. In the case of 4MRs, the bond length within the Be_12_O_12_ nanocage measures 1.57 Å. Within the 6MRs the distinct bond lengths are observed to be 1.51 Å, 1.52 Å, and 1.58 Å respectively. These results closely align with the findings of Mashahdzadeh and colleagues who previously reported bond lengths of 1.587 and 1.662 Å between the beryllium atom and oxygen atom in the 6MRs and 4MRs respectively^57^.

The MEP map as shown in Fig. 1 serves the purpose of identifying potential interaction sites by detecting areas of electron-rich and electron-deficient centers based on the coloration around the atoms. Using the MEP map, we optimized various complexes involving Be_12_O_12_ and paracetamol. This was done to determine the most favorable interaction sites. Notably, the MEP map highlights an electrophilic center through yellow and red shading around the N atom within the ring, as well as around the O atom in the O–H bond and the C=O group. Similarly, the nucleophilic nature of the blue-shaded regions surrounding H atoms attached to carbons in paracetamol is observed. Additionally, the MEP map of Be_12_O_12_ reveals blue shading around boron atoms, while Be atoms are encompassed by yellow–red hues, indicating their potential for complex formation.

**Figure 2:**
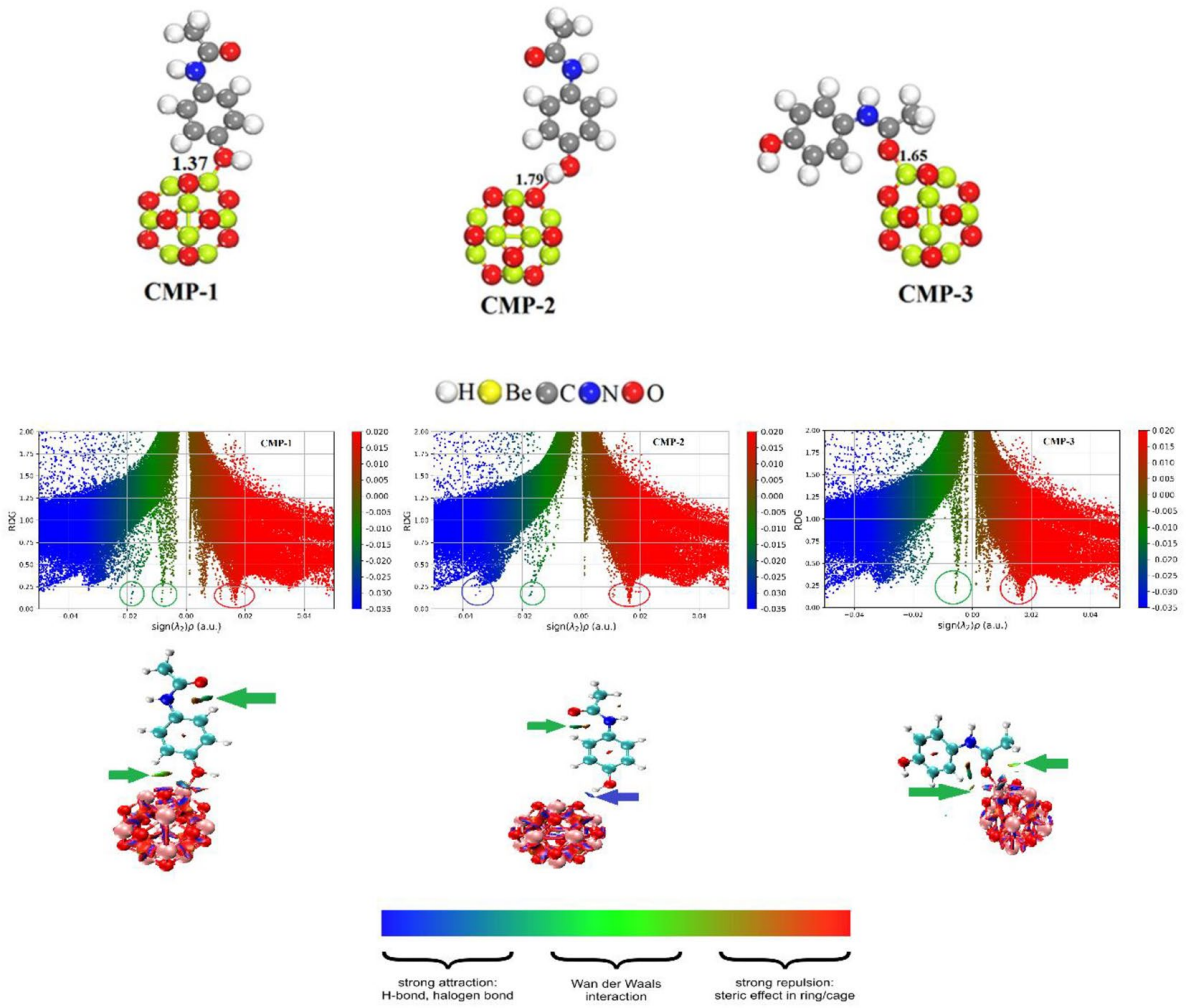
Optimized complexes, RDG and NCI study of the complexes of Be_12_O_12_ nanocage with paracetamol.

The adsorption capability of the Be_12_O_12_ nanocage concerning paracetamol adsorption has been assessed, considering various configurations. Each geometry was separately optimized. The collection of the most stable complexes between Be_12_O_12_ and paracetamol is presented in Table 1, along with their respective most stable structures and corresponding *E*_ads_. These complex structures, along with bond length information, are illustrated in Fig. 2. The interactions between paracetamol molecules and Be atoms within the nanocage predominantly occur through the oxygen atom of the C=O group and the oxygen atom of the O–H group. Additionally, weaker interactions transpire between paracetamol molecules and the oxygen atom of the nanocage via the hydrogen atom of the O–H bond. CMP-1 and CMP-3 exhibited *E*_ads_ values exceeding −11 kcal/mol in the gas phase, indicating chemisorption, as detailed in Table 1. However, in the case of CMP-3, *E*_ads_ values were below −11 kcal/mol in the gas phase, suggesting physisorption.

**Table 1:** Frontier molecular orbital (FMO), charge transfer analysis and Adsorption energies (*E*_ads_) of the optimized complexes of Be_12_O_12_ with paracetamol.

System	*E*_ads_ (Kcal/mol) 6-31G (d, p)	*E*_ads_ (Kcal/mol) Aug-cc-pvdz	E_LUMO_ (eV)	E_HOMO_ (eV)	E_g_ (eV)	Charge transfer (e)
Be_12_O_12_	–	–	− 0.25524	− 8.45349	8.198251	–
Paracetamol	–	–	− 0.08327	− 5.47929	5.396021	–
CMP-1	− 23.4333	− 13.5084	− 0.6694	− 6.23223	5.562827	− 0.112
CMP-2	− 10.4163	− 3.6205	− 0.44627	− 5.31602	4.869752	0.021
CMP-3	− 31.2316	− 23.4103	− 1.1807	− 6.23576	5.055062	− 0.159

The interactions in CMP-1 and CMP-3 predominantly involve electrostatic forces, while CMP-2 features an additional H-bond interaction. The robust nature of the interaction in CMP-1 and CMP-3 is also evident from their notably compact bond lengths of 1.37 and 1.65 Å, respectively. On the other hand, the bond length in CMP-2 measures 1.79 Å, which is responsible for the H-bond interaction. CMP-3 displayed the highest *E*_ads_ values, indicating the most stable geometry among the three, followed by CMP-1 and CMP-2. Next the effect of larger basis set i.e., aug-cc-pvdz was tested on the *E*_ads_ values and found that the *E*_ads_ values decrease to some extent, however the order of the strength of interaction suggested by B3LYP/6-31G(d,p) basis set is replicated by B3LYP/aug-cc-pvdz. The study at higher larger basis set further evidences the presence of stronger interaction in CMP-3 and CMP-1 as compared to CMP-2.

In order to comprehend the interaction between the Be_12_O_12_ nanocage and paracetamol, an RDG analysis was conducted. Figure 2 illustrates the RDG plot in relation to sign(λ2)ρ, along with the NCI plot^58^. These analytical tools hold substantial importance in examining both attraction and repulsion phenomena between the Be_12_O_12_ nanocage and paracetamol. Furthermore, the RDG analysis proves valuable in distinguishing between robust and subtle interactions between the two entities. The nature of interaction is contingent upon the positions and characteristics of distinct peaks in the RDG plot, as well as the varied colors depicted in the NCI plot. Within the NCI plot, the presence of a blue region signifies the occurrence of hydrogen bonding, while a green area suggests a weaker van der Waals interaction. Conversely, the presence of a red zone indicates a substantial repulsion or steric interaction. The arrangement of distinct peaks within the RDG isosurface, along with values of (sign λ2)ρ < 0, corresponds to diverse interaction types. Notably, in the RDG plots of CMP-1 and CMP-3, the spikes encircled with green color depicts electrostatic interactions. These interactions occur between the hydrogen atom of paracetamol and the oxygen atom of the nanocage. Conversely, the presence of a red encircled spike indicates repulsion occurring at various positions. Lastly, in CMP-2, a blue encircled spike illustrates a hydrogen bond formation between the hydrogen atom of the O–H bond and the oxygen atom of the nanocage and green encircled spike demonstrate intramolecular electrostatic interaction in paracetamol molecule, as depicted in Fig. 2. The NCI plots of the complexes effectively unveil the interactions, showcasing diverse types indicated by differently colored arrows connecting the interacting atoms. Likewise, in CMP-1 and CMP-3, the green arrows indicating interactions between the hydrogen atom of paracetamol and the oxygen atom of the nanocage, underscore strong electrostatic interactions. Intramolecular electrostatic interaction also exists in CMP-1 which is shown by green encircled spike and green arrow in Fig. 2. In CMP-3, the extensive green region at different position signifies intensified electrostatic interactions along with strong bond between oxygen atom of paracetamol and beryllium atom of the nanocage, correlating with elevated *E*_ads_ values despite of the smaller bond length values from CMP-1.

Further, a blue arrow highlights interaction through H-bonding, depicted by the blue region between the hydrogen atom of O–H and the oxygen atom of the nanocage. The presence of repulsion is discerned by red colors between interacting atoms in various positions across the nanocage and paracetamol. The stable geometry and chemisorbed characteristics of CMP-1 and CMP-3 result from the presence of two robust electrostatic bonds and interactions at different positions. Analyzing using RDG and NCI, it becomes evident that the Be_12_O_12_ nanocage forms a strong interaction with the paracetamol drug.

### Natural bond orbitals (NBO), quantum theory of atoms in molecule (QTAIM) and Frontier molecular orbitals (FMO) Analyses

The investigation into charge transfer utilized NBO analysis^59^. The optimized configurations of Be_12_O_12_ complexes with paracetamol were assessed using B3LYP/6-31G (d,p) theory level. The findings demonstrated negative charges on the O atom of the carbonyl groups and the O–H bond in both paracetamol and Be_12_O_12_ nanocage. Conversely, positive charges were observed on the H and Be atoms in paracetamol and Be_12_O_12_ nanocage.

The charge on the O atom within the O–H bond in the paracetamol molecule (CMP-1) increased from − 0.694 to − 0.806 eV, implying a charge transfer of − 0.112 eV from the nanocage to paracetamol. In CMP-2 and CMP-3, the charge on the H atom of the O–H bond and the O atom of the carbonyl group increased from 0.491 and − 0.625 eV to 0.512 and − 0.784 eV, resulting in charge transfers of 0.021 and − 0.159 eV, respectively. Notably, the most substantial charge transfer was observed in CMP-3, justifying the highest adsorption energies values and indicative of robust chemisorption. This result aligns with the *E*_ads_ and justifying the higher bond length values of CMP-3 with the highest adsorption energies values. CMP-1 also exhibited considerable charge transfer, suggesting a strong interaction and chemisorption. In contrast, CMP-2 displayed the smallest *E*_ads_, indicating physisorption, which is further supported by the minimal charge transfer value. Based on NBO analysis, Be_12_O_12_ nanocage emerges as a promising candidate for paracetamol removal from wastewater.

**Figure 3:**
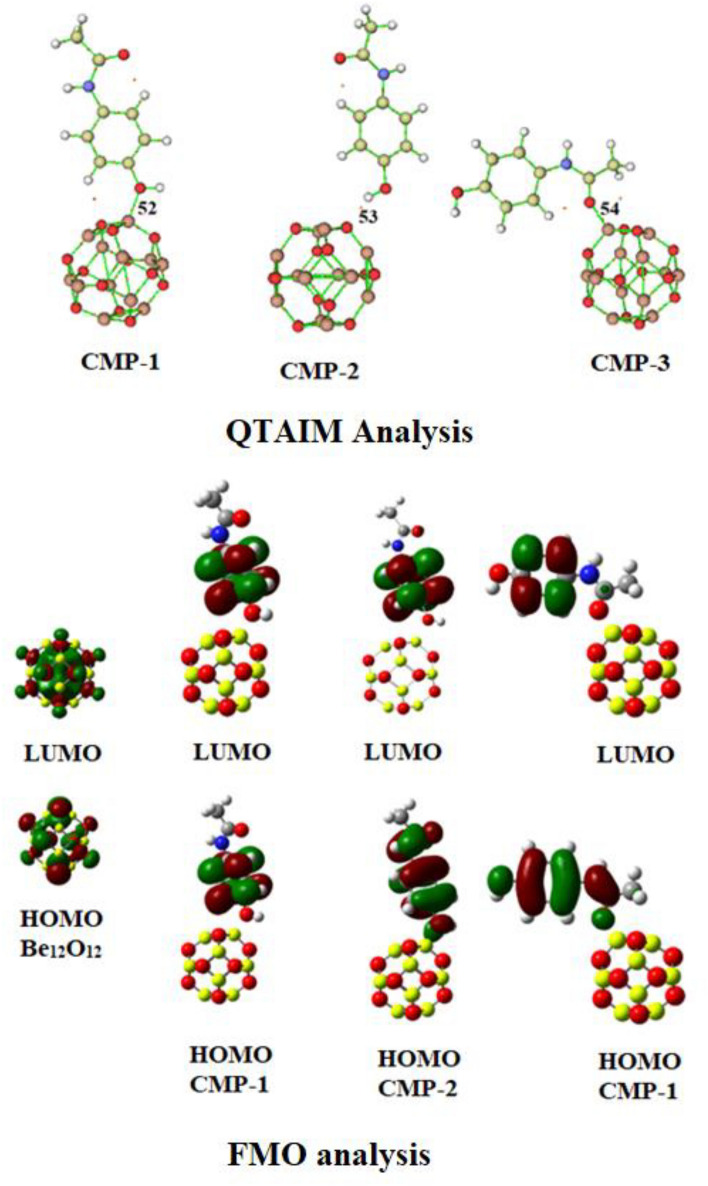
QTAIM and FMO investigation of complexes of Be_12_O_12_ nanocage with paracetamol.

The interaction between Be_12_O_12_ nanocage and paracetamol molecules was thoroughly examined through QTAIM analysis. The Gaussian program was used to carried out this study, simulating at the B3LYP/6-31G (d,p) level of theory. The bond critical points are illustrated in Fig. 3. The study examined various topological parameters at the BCP (Bond Critical Point) to characterize both covalent and weak electrostatic interactions resulting from electron sharing or transferring^60^. Weak electrostatic interaction becomes evident when both H_b_ and the Laplacian of electron density (∇^2^ρ_b_) are positive. Conversely, negative H_b_ and ∇^2^ρ_b_ values signify covalent interactions. The coexistence of partial covalent and electrostatic interactions is indicated when Hb is negative and ∇^2^ρ_b_ is positive.

Additionally, interactions can be categorized as weak electrostatic and non-covalent when the ratio of -G_b_/V_b_ exceeds 1, whereas strong covalent interactions are indicated by values less than 1. Partial interactions are suggested for values between 0.5 and 1.

The data provided in Table 2 concerning the nanocage under investigation indicates that electrostatic interactions exist in CMP-1 and CMP-3 between paracetamol and Be_12_O_12_. This deduction is drawn based on the positive H_b_ and ∇^2^ρ_b_ values observed at BCP 52 and 54, coupled with the identification of a partial interaction comprising both covalent and electrostatic interaction in CMP-2 at BCP 53 as cleared from the negative values of H_b_ and positive values of ∇^2^ρ_b_. This notion is further supported by the values of -Gb/Vb, which is between 0.5 and 1 at BCP 53 and exceed unity at BCP 52 and 54.

**Table 2:** QTAIM study of complexes of Be_12_O_12_ nanocage with paracetamol.

Complex	BCP	ρ_b_ (au)	∇^2^ρ_b_ (au)	G_b_ (au)	V_b_ (au)	H_b_ (au)	G_b_/V_b_ (au)
CMP-1	52	0.04907	0.35364	0.08160	− 0.07480	0.00680	1.09
CMP-2	53	0.03608	0.10893	0.02753	− 0.02784	− 0.00030	0.98
CMP-3	54	0.06070	0.46558	0.10653	− 0.09666	0.00986	1.10

The most notable density values of ρ_b_ can be found at BCP 54 within CMP-3, demonstrating the strength of this particular bond compared to others. This trend is followed by BCP 52 within CMP-1. Noteworthy is the relatively lower ρ_b_ value at BCP 53, alongside with the small positive ∇^2^ρ_b_ values indicating weak hydrogen bond. These findings collectively suggest a significant interaction between paracetamol and the Be_12_O_12_ nanocage, facilitated by hydrogen bonding.

The adsorption characteristics of paracetamol molecules onto the Be_12_O_12_ nanocage were additionally explored through FMO analysis. phenomenon further amplifies the nanocage's ability to adsorb paracetamol molecules^61^. The values associated with E_HOMO_ and E_LUMO_ can be found in Table 1. A noticeable reduction was observed in the EHOMO (HOMO energy) values, coupled with a significant increase in the E_LUMO_ (LUMO energy) values. This led to a notable decrease in the HOMO–LUMO energy gap. Evidently, the bandgap values decreased for all three complexes, underscoring a strong interaction between paracetamol and the Be_12_O_12_ nanocage. The reduction in energy gap within all complexes also provides strong support for increased charge transfer. In DFT calculations, these molecular orbitals are commonly referred to as Kohn–Sham orbitals. Utilizing the Koopmans theorem, these values offer insights into electron affinity (A = − E_LUMO_) and ionization energy (I = − E_HOMO_) computations. Consequently, our calculations reveal that as electron affinity rises, ionization energy diminishes. Within this investigation, the primary focus lies on the O and Be atoms of the nanocage in relation to the HOMO and LUMO. Notable alterations occur following complex formation, with the LUMO and HOMO becoming predominantly associated with paracetamol, indicating robust interactions. Specifically, in CMP-1 and CMP-3, the HOMO exhibits proximity to the interaction site compared to CMP-2, showcasing intensified interactions in CMP-1 and CMP-3. The proximity of the LUMO and HOMO to the interacting atoms implies substantial charge transfer, underscoring pronounced interactions. These findings align with the NBO analysis.

### Electron density difference (EDD), polarizable continuum model, dipole moment, thermodynamics, regeneration and molecular dynamics study

**Figure 4:**
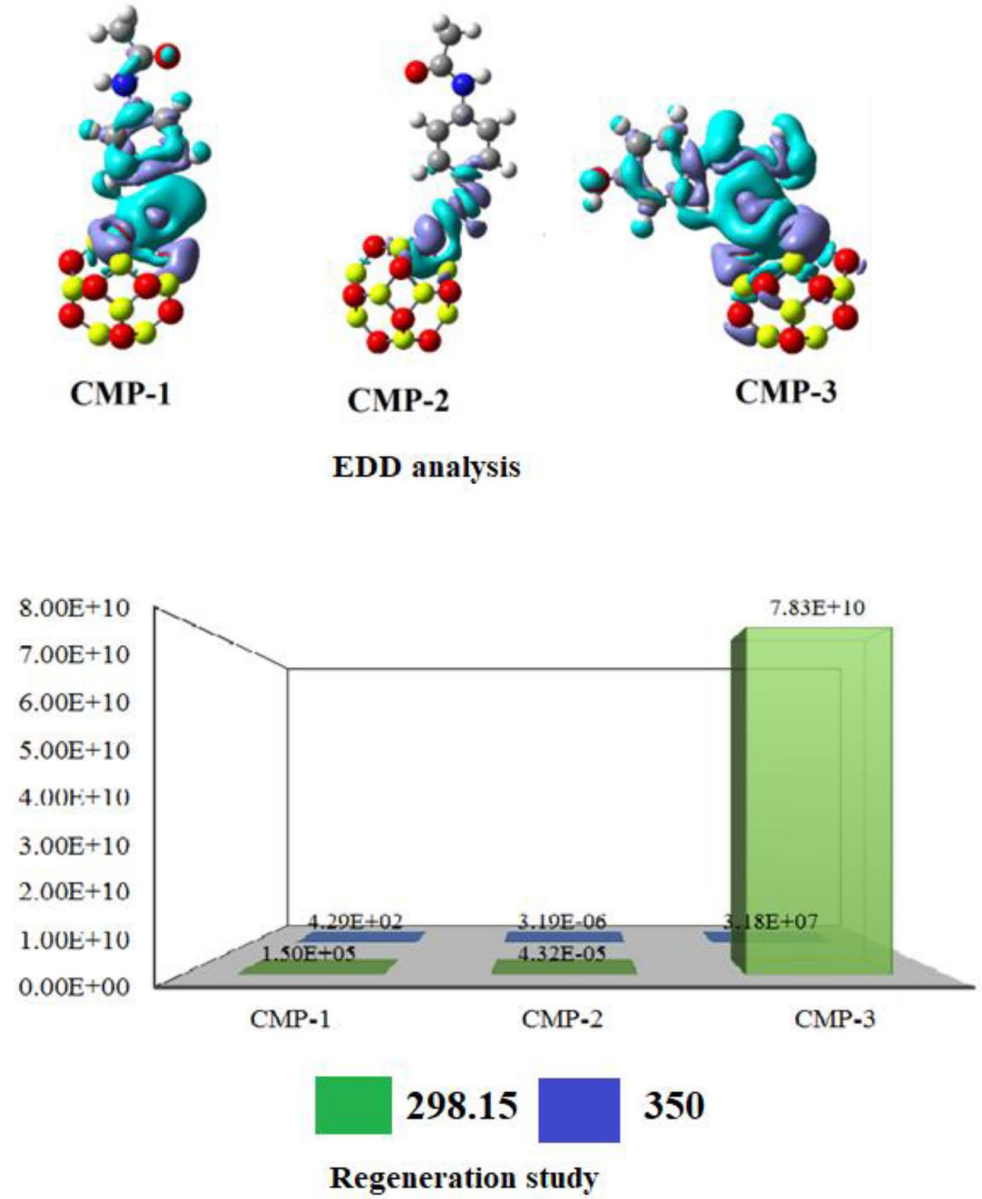
EDD and Recovery time graph of complexes of Be_12_O_12_ nanocage with paracetamol.

The analysis of Electron Density Difference (EDD) is utilized to investigate orbital interaction and validate the transfer of charge between the adsorbent and adsorbate. This method was applied to visualize the regions of atoms in interaction and the overlapping orbitals. Figure 4 displays electron density difference isosurfaces. This analysis accounts for electron density variations in complexes compared to the sum of adsorbent and adsorbate, specifically focusing on the interaction between paracetamol drug and Be_12_O_12_ nanocage. EDD isosurfaces show blue areas indicating charge accumulation and green regions signifying charge depletion during the paracetamol drug adsorption onto the Be_12_O_12_ nanocage. Strong interactions with maximum charge transfer are indicated by the presence of green and blue isosurfaces between interacting atoms across all complexes. Furthermore, in CMP-1 and CMP-3, the substantial overlap of continuous blue and green loops, without any gaps at the interaction sites, underscores their strong interactions. In contrast, the smaller sized blue and green loops at the interaction sites of CMP-2 suggest weaker interactions in this complex.

To analyze the impact of water on the adsorption process mechanism, we investigated the interaction between a B_12_O_12_ nanocage and paracetamol in an aqueous environment. This was accomplished using the polarizable continuum model (PCM)^42^. The resulting adsorption data were compiled and documented.

Following solvation, the adsorption values for CMP-1, CMP-2, and CMP-3 exhibit a minor decrease, while the nature of interaction remains largely unaltered. In an aqueous environment, CMP-1 and CMP-3 demonstrate chemisorption characteristics, whereas CMP-2 displays physisorption behavior, as evidenced by the *E*_ads_ values. Notably, in water, CMP-3 displays the highest adsorption energy at approximately − 27.8869 kcal/mol, underscoring its structural stability both in gaseous and aqueous mediums. Likewise, we tested the effect of dimethyl sulfoxide (DMSO) on the process of adsorption which affected the adsorption energies of CMP-2 and CMP-3 to a great extent, however CMP-1 and CMP-3 show chemisorption and CMP-2 depicts physisorption in DMSO which is consistent with aforementioned results.

The verification of interaction was also confirmed through dipole moment analysis. Notably, there was a noticeable rise in dipole moment (DM) values within the complexes formed by Be_12_O_12_ and paracetamol. The dipole moment registered for Be_12_O_12_ nanocage stood at 0.002387 Debye, and this value exhibited further augmentation in the case of CMP-1, CMP-2, and CMP-3. This trend strongly indicates a substantial interaction between the adsorbent and the adsorbate. The most significant increase in dipole moment was observed in CMP-3, with a remarkable value of approximately 8.132803 Debye. These escalated DM values for the complexes provide additional confirmation of Be_12_O_12_ nanocage's exceptional ability to efficiently adsorb paracetamol molecules from aqueous solutions.

Thermodynamic analysis was conducted to assess the viability and characterize the process nature. The computed ∆H and ∆G values for the CMP-1, CMP-2, and CMP-3 complexes were all found to be negative, indicating an exothermic interaction, as outlined in Table 3. These findings indicate both the feasibility and spontaneous occurrence of the process, aligning with the energetic assessments.

**Table 3:** Analysis of Be_12_O_12_ complexes with paracetamol: adsorption energy (*E*_ads_) Comparison in gas and aqueous phases, dipole moment, and thermodynamics study.

System	*E*_ads_(water medium) (Kcal/mol)	*E*_ads_(DMSO) (Kcal/mol)	DM (Debye)	∆H (Kcal/mol)	∆G (Kcal/mol)
Be_12_O_12_	–	–	0.002387	–	–
CMP-1	− 20.9023	− 23.9165	5.224273	− 18.34	− 16.93
CMP-2	− 8.62381	− 4.99724	5.221608	− 5.21	− 3.06
CMP-3	− 27.8869	− 13.7587	8.132803	− 26.54	− 23.76

An effective adsorbent should possess the ability to be reclaimed efficiently, ensuring swift recovery. We computed the recovery times for all complexes at both 298.15 K and 350 K temperatures, as demonstrated in Fig. 4. The recovery duration of the Be_12_O_12_ adsorbent within CMP-1, CMP-2, and CMP-3 at 298.15 K is projected at 1.50 × 10^5^ s, 4.32 × 10^–5^ s, and 7.83 × 10^10^ s, respectively. At 350 K, these times become 4.29 × 10^2^ s, 3.19 × 10^–6^ s, and 3.18 × 10^7^ s, respectively. This suggests that the Be_12_O_12_ adsorbent is suitable for paracetamol removal from wastewater due to its rapid regeneration at ambient temperature. These recovery time findings underscore the Be_12_O_12_ nanocage's superiority in eliminating paracetamol from potable water. The overall study disclosed that the adsorbent is extremely effective, regenerable and reusable both in gas and aqueous phase and offering a practical avenue for efficiently purifying water from emerging pollutants in future.

**Figure 5:**
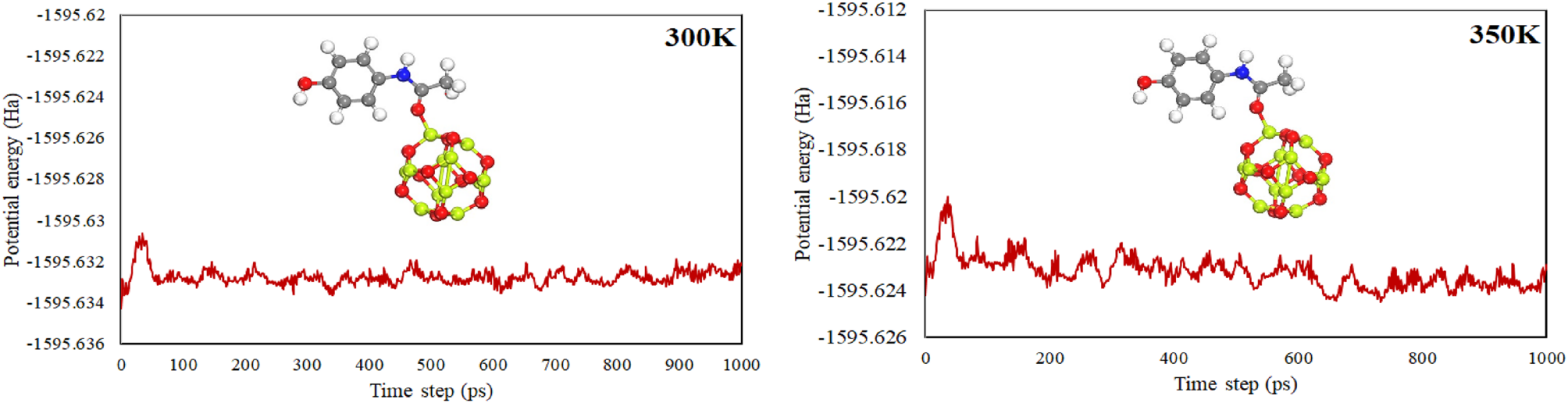
AIMD study of complexes of Be_12_O_12_ nanocage with paracetamol.

The stability of the complexes was studied using CMP-3 as a reference at two different temperature employing Ab-initio molecular dynamics simulations (AIMS). The AIMD simulations were performed at two different temperature i.e., 300 K and 350 K for 1 ps with a time step of 1 fs. The structures of CMP-3 and potential energy at 300 K and 350 K as illustrated in Fig. 5. The AIDM study discloses that no distortion occurred in the structure of nanocage and paracetamol drug and the drug still exist at the interactive position with the variation of potential energy at certain magnitude. The study demonstrated that the nanocage is effective to interact and adsorb paracetamol at higher temperature and the complexes are stable and the adsorbate will remain bonded on the surface.

The overall research evaluated stronger interaction in CMP-1 and CMP-3 in both aqueous and gas environments, as evidenced by *E*_ads_ values exceeding −11 kcal/mol. Conversely, CMP-2 exhibited weak interaction as evident from its *E*_ads_ values falling below −11 kcal/mo. Through reduced density gradient (RDG) and non-covalent interactions (NCI) analyses, we established that robust bonds exist between CMP-1 and CMP-3 of an electrostatic nature, while a weak hydrogen bond is present in CMP-2. Natural orbital analysis showed highest charge transfer in CMP-3 justifying its higher *E*_ads_ values than CMP-1 despite of larger bond length. Quantum theory of atoms in molecules (QTAIM) analysis revealed electrostatic nature in CMP-1 and CMP-3, while CMP-2 exhibited shared interactions. Frontier molecular theory (FMO) analysis indicated a decrease in bandgap across all complexes, signifying Be_12_O_12_ nanocage's efficacy in paracetamol elimination from wastewater. Dipole moment analysis highlighted diminished dipole moment, showcasing the Be_12_O_12_ nanocage's potential for drug adsorption. Electron density difference (EDD) analysis demonstrated considerable orbital overlap between paracetamol and Be_12_O_12_ nanocage. Thermodynamic evaluation confirmed process feasibility and spontaneity, indicated by negative free energy values. The application of polarizable continuum model (PCM) revealed the nanocage's optimal performance in both gas and aqueous as well as in non-aqueous mediums, making it a promising solution for decontaminating antibiotic-polluted water. Our regeneration study underscored the adsorbent's facile recovery, offering a practical avenue for efficiently purifying water from emerging pollutants in the future.

## Methods and tools

### Computational simulations and analyses details

The structures of adsorbent and adsorbate were built using free available software Avogadro which is an advanced chemical editor and were initially optimized using the auto optimization tool^62^. The input file for each individual monomer was prepared in Avogadro software using the tight convergence option and optimized using GAMESS US program^63^ employing the B3LYP theory with a 631-G(d,p) basis set. Following the same procedures, the complexes between the adsorbent and the drug were generated using the Avogadro software based on the optimized monomer geometries. Subsequent optimization of these complexes was performed using the GAMESS US program. To enhance accuracy, the DFT-D3 (dispersion correction) scheme developed by Grimme^64^ was applied to adjust the adsorption energies of all complexes. The determination of adsorption energy for each complex was executed through the utilization of the following formula:

1 $$E_{ads} = E_{complex} - \left( {E_{nanocage} + E_{paracetamol} } \right),$$

where *E*_complex_ denotes the overall electronic energy of the optimized complex system, and *E*_nanocage_ and *E*_paracetamol_ corresponds to the total electronic energies of the individual monomers, namely the paracetamol and nanocage.

The investigation of strong or weak interactions between the adsorbents and adsorbates was conducted through reduced density gradient (RDG) analysis using the Multiwfn code^65^. To detect the presence of hydrogen bonds and electrostatic interaction between adsorbate and adsorbents, non-covalent interaction (NCI) analysis was employed, utilizing the VMD software^66^ and the colored RGD figures were obtained using the free online site^67^. For assessing charge transfer, natural bonding orbitals (NBO) analysis was performed using the NBO code within Gaussian 16^68^. Quantum theory of atoms in molecules (QTAIM) analysis was adopted to explore interaction strength and behavior of interaction between paracetamol and nanocage^60^. Extracted from B3LYP/631-G(d,p) simulations, the wavefunction provided crucial data for determining various topological parameters at bond critical points (BCPs), including laplacian (∇^2^ρ_b_), total electron energy densities (H_b_), electron densities (ρ_b_), kinetic electron density (G_b_), and potential electron energy density (V_b_), utilizing Gaussian 16 programs^68^.

The sensing ability evaluation of adsorbents for adsorbates was performed using frontier molecular orbitals (FMO) analysis. Bandgap (Eg) values for all complexes were calculated according to the equation,

2 $$E_{g} = E_{LUMO} - E_{HOMO} ,$$

where *E*_g_ signifies the energy gap or bandgap, while LUMO and HOMO represent the lowest unoccupied molecular orbitals and highest occupied molecular orbitals, respectively.

Visualization of orbital overlap was simulated via energy density difference (EDD) analysis using both Multiwfn software and Gaussian 16. To assess the impact of the aqueous medium on the adsorption process, the polarizable continuum model (PCM) implemented in the GAMESS UK code was utilized^63^. The feasibility and nature (spontaneous/nonspontaneous) of the process were evaluated through thermodynamics simulations. To investigate changes in free energy (ΔG) and enthalpy (ΔH), the following equations were employed:

3 $$\Delta H_{ads} = H_{Complex} - H_{{\left( {nanocage + paracetamol} \right)}} ,$$

4 $$\Delta G_{ads} = \Delta H_{ads} - T\Delta S_{ads} ,$$

5 $$\Delta G_{ads} = \Delta H_{ads} - T[\left( {S_{{\left( {complex} \right)}} - \left( {S_{nanocage} + S_{paracetamol} } \right)} \right],$$

In this context, H signifies the collective electronic and thermal enthalpy, G represents the combined electronic and thermal Gibbs free energy, and S denotes the entropy at 298.15 K and 1 atm.

The assessment of adsorbent regeneration (recovery time) within all complexes was conducted employing the subsequent equation.

6 $$\tau = \nu_{0}^{ - 1} \exp \left( { - E_{ads} /kT} \right),$$

Here, $\nu_{0}^{ - 1}$ denotes the attempt frequency, *E*_ads_ signifies the adsorption energy, k stands for Boltzmann's constant, and T represents the temperature^69^.

The stability of the complexes was studied using the CMP-3 as a reference employing Ab-initio molecular dynamics (AIMD) simulations with the help of DMOl3 code^70^. The AIMD simulations were achieved via PBE functional with the generalized gradient approximation (GGA). The basis set used is double numerical plus polarization (DNP). Thermal smearing parameter was established to 0.005 au and basis set cutoff was set to 4.6 Å^71,72^.

### Supplementary Information

Supplementary Information.

## Data Availability

All data generated or analyzed during this study are included in this published article and its supplementary information file.
