# Supplementary material for: Exploring the promising application of Be12O12 nanocage for the abatement of paracetamol using DFT simulations
Source: Sci Rep. 2023 Oct 28;13:18481. doi: 10.1038/s41598-023-45674-3 (PMC10613287; doi:10.1038/s41598-023-45674-3)
Supplement: Supplementary file 1 — Supplementary Information. [file 41598_2023_45674_MOESM1_ESM.docx]

**Coordinates of the optimized nanocage, paracetamol and their complexes**

Be_12_O_12_

Be 4.0 -3.27330 1.59799 2.28149

O 8.0 -4.18288 0.49802 2.94816

Be 4.0 -5.27589 1.60815 2.72986

O 8.0 -4.31381 2.78341 2.29614

Be 4.0 -4.40119 -0.70201 2.04650

Be 4.0 -4.59133 3.38376 0.93214

O 8.0 -2.23643 1.35591 1.18846

O 8.0 -6.65745 1.38555 2.11966

Be 4.0 -2.57052 0.04544 0.36070

O 8.0 -3.41937 -1.10985 0.86783

O 8.0 -5.75987 -1.10603 1.37010

Be 4.0 -6.70877 0.07501 1.23696

Be 4.0 -6.82027 2.06285 0.70285

O 8.0 -5.97142 3.21814 0.19572

O 8.0 -3.63092 3.21432 -0.30655

Be 4.0 -2.68202 2.03329 -0.17341

O 8.0 -2.73334 0.72274 -1.05611

Be 4.0 -4.79946 -1.27546 0.13141

O 8.0 -7.15436 0.75238 -0.12491

Be 4.0 -4.98960 2.81030 -0.98295

O 8.0 -5.07698 -0.67512 -1.23259

Be 4.0 -6.11750 0.51030 -1.21794

O 8.0 -5.20792 1.61027 -1.88461

Be 4.0 -4.11490 0.50014 -1.66631

Paracetamol

C 6.0 -1.23961 3.44671 0.00033

C 6.0 -0.11554 2.60619 -0.00133

C 6.0 -0.30822 1.21717 0.00015

C 6.0 -1.60212 0.69722 0.00205

C 6.0 -2.71586 1.54015 0.00043

C 6.0 -2.52842 2.92648 0.00088

O 8.0 -4.00509 1.07824 -0.00602

H 1.0 -3.99282 0.11282 0.00059

N 7.0 1.16025 3.21419 -0.00184

C 6.0 2.40233 2.62450 0.00103

H 1.0 1.14805 4.22787 0.00385

O 8.0 2.58717 1.41319 0.00123

C 6.0 3.56879 3.60171 -0.00087

H 1.0 3.27896 4.65533 0.00200

H 1.0 4.18582 3.40527 -0.88299

H 1.0 4.18785 3.40192 0.87968

H 1.0 -1.10593 4.52720 -0.00147

H 1.0 -3.39593 3.57711 0.00294

H 1.0 0.55156 0.56324 -0.00335

H 1.0 -1.74002 -0.38207 0.00273

CMP-1

Be 4.0 -2.80243 -3.01377 -0.86660

O 8.0 -2.43426 -4.24153 0.05181

Be 4.0 -2.35562 -3.07549 1.11462

O 8.0 -2.45035 -1.85537 0.13724

Be 4.0 -3.59008 -5.20372 0.23192

Be 4.0 -3.59521 -0.80205 0.40438

O 8.0 -3.96702 -2.99256 -1.83713

O 8.0 -2.95544 -3.20686 2.51279

Be 4.0 -5.10015 -4.05344 -1.55114

O 8.0 -4.82815 -5.31705 -0.74269

O 8.0 -4.34023 -5.40622 1.59928

Be 4.0 -4.13967 -4.24593 2.56449

Be 4.0 -4.17222 -2.21193 2.65498

O 8.0 -4.40737 -0.98423 1.81880

O 8.0 -4.95337 -0.82402 -0.51141

Be 4.0 -5.13102 -2.00958 -1.43689

O 8.0 -6.31559 -3.07042 -1.39439

Be 4.0 -5.58712 -5.28012 0.63662

O 8.0 -5.30986 -3.27877 2.95791

Be 4.0 -5.62938 -1.11681 0.86368

O 8.0 -6.82047 -4.44128 0.96943

Be 4.0 -6.47148 -3.26549 1.98713

O 8.0 -6.81948 -2.05273 1.05559

Be 4.0 -6.93178 -3.20434 -0.00479

C 6.0 -4.83318 3.63649 0.92122

C 6.0 -3.87858 4.65800 1.05266

C 6.0 -2.51517 4.33088 0.99546

C 6.0 -2.13102 3.00821 0.76823

C 6.0 -3.09534 2.02148 0.60367

C 6.0 -4.45177 2.32087 0.68425

O 8.0 -2.73616 0.70282 0.31711

H 1.0 -1.77860 0.55859 0.28172

N 7.0 -4.35253 5.97625 1.22519

C 6.0 -3.64263 7.15749 1.14381

H 1.0 -5.35619 6.06158 1.29665

O 8.0 -2.42728 7.21677 1.03355

C 6.0 -4.49871 8.41586 1.18724

H 1.0 -5.55956 8.24030 1.38719

H 1.0 -4.40989 8.92377 0.22011

H 1.0 -4.09391 9.08183 1.94959

H 1.0 -5.89289 3.87396 0.97705

H 1.0 -5.18246 1.53852 0.53061

H 1.0 -1.77434 5.10927 1.10435

H 1.0 -1.07291 2.75973 0.72102

CMP-2

Be 4.0 -3.06025 -3.36765 2.44536

O 8.0 -4.00496 -4.59822 2.67873

Be 4.0 -5.06452 -3.43384 2.78754

O 8.0 -4.08552 -2.20432 2.77000

Be 4.0 -4.14024 -5.45909 1.43244

Be 4.0 -4.28798 -1.21652 1.63756

O 8.0 -1.99152 -3.24304 1.36694

O 8.0 -6.40600 -3.42714 2.06217

Be 4.0 -2.25974 -4.23842 0.17578

O 8.0 -3.11853 -5.50223 0.23334

O 8.0 -5.48894 -5.57162 0.61452

Be 4.0 -6.45296 -4.41627 0.84042

Be 4.0 -6.49421 -2.37395 0.88274

O 8.0 -5.63709 -1.10407 0.82990

O 8.0 -3.26200 -1.02062 0.45319

Be 4.0 -2.29614 -2.19869 0.22876

O 8.0 -2.35008 -3.17608 -0.99568

Be 4.0 -4.47560 -5.39073 -0.57209

O 8.0 -6.75630 -3.37328 -0.30411

Be 4.0 -4.62601 -1.13629 -0.37477

O 8.0 -4.67369 -4.40076 -1.71250

Be 4.0 -5.69130 -3.23678 -1.38295

O 8.0 -4.74206 -2.00538 -1.60954

Be 4.0 -3.68779 -3.17723 -1.72403

C 6.0 -0.37256 4.38988 0.05071

C 6.0 -1.51409 5.05392 0.52054

C 6.0 -2.63842 4.29299 0.85653

C 6.0 -2.60395 2.90118 0.73242

C 6.0 -1.46731 2.23828 0.25917

C 6.0 -0.35128 3.00966 -0.07662

O 8.0 -1.38252 0.88363 0.13430

H 1.0 -2.22509 0.43465 0.34122

N 7.0 -1.46411 6.46508 0.59976

C 6.0 -2.45651 7.35101 0.94470

H 1.0 -0.58462 6.87339 0.31359

O 8.0 -3.58083 7.03727 1.31106

C 6.0 -2.07628 8.81931 0.80192

H 1.0 -1.01451 8.99409 0.59743

H 1.0 -2.33998 9.34763 1.71822

H 1.0 -2.65654 9.28761 0.02111

H 1.0 0.50581 4.97966 -0.20294

H 1.0 0.52764 2.50665 -0.45187

H 1.0 -3.52435 4.79442 1.22434

H 1.0 -3.47664 2.32418 1.02649

CMP-3

Be 4.0 -2.60786 -4.16858 -0.85361

O 8.0 -2.45972 -5.41161 0.09585

Be 4.0 -2.25409 -4.24088 1.15464

O 8.0 -2.12036 -3.04060 0.13671

Be 4.0 -3.78243 -6.12229 0.33009

Be 4.0 -3.21599 -1.99037 0.35524

O 8.0 -3.69725 -3.98184 -1.90059

O 8.0 -2.91501 -4.17503 2.51310

Be 4.0 -4.97299 -4.84177 -1.54986

O 8.0 -5.00833 -6.08528 -0.66980

O 8.0 -4.58708 -6.18672 1.68247

Be 4.0 -4.26084 -4.99840 2.57325

Be 4.0 -3.95979 -2.96974 2.58058

O 8.0 -3.91344 -1.72871 1.72911

O 8.0 -4.33801 -1.57978 -0.64977

Be 4.0 -4.67557 -2.79200 -1.49139

O 8.0 -6.00951 -3.65572 -1.46677

Be 4.0 -5.77364 -5.84584 0.69724

O 8.0 -5.23288 -3.82898 2.95554

Be 4.0 -5.22490 -1.42743 0.77331

O 8.0 -6.83264 -4.78679 0.91521

Be 4.0 -6.31160 -3.64548 1.89263

O 8.0 -6.44200 -2.43226 0.94585

Be 4.0 -6.64651 -3.57190 -0.09802

C 6.0 -8.89978 3.04843 1.36814

C 6.0 -8.07894 1.91864 1.23246

C 6.0 -8.62395 0.64372 1.44794

C 6.0 -9.96899 0.52296 1.79286

C 6.0 -10.78350 1.65163 1.92642

C 6.0 -10.23947 2.92304 1.71156

O 8.0 -12.10499 1.58436 2.25729

H 1.0 -12.36107 0.66045 2.37971

N 7.0 -6.72083 2.15099 0.85927

C 6.0 -5.71848 1.26411 0.73070

H 1.0 -6.49243 3.11388 0.65728

O 8.0 -5.91156 0.06368 0.99579

C 6.0 -4.37222 1.74224 0.26141

H 1.0 -4.31904 2.82045 0.08805

H 1.0 -4.12616 1.19642 -0.65577

H 1.0 -3.63097 1.45043 1.01187

H 1.0 -8.48824 4.04240 1.20456

H 1.0 -10.87525 3.79421 1.81845

H 1.0 -8.01692 -0.24635 1.34831

H 1.0 -10.38253 -0.46875 1.95947
